# Obesity accelerates epigenetic aging in middle-aged but not in elderly individuals

**DOI:** 10.1186/s13148-016-0301-7

**Published:** 2017-02-14

**Authors:** Tapio Nevalainen, Laura Kananen, Saara Marttila, Juulia Jylhävä, Nina Mononen, Mika Kähönen, Olli T. Raitakari, Antti Hervonen, Marja Jylhä, Terho Lehtimäki, Mikko Hurme

**Affiliations:** 1 Department of Microbiology and Immunology, School of Medicine, University of Tampere, Tampere, Finland; 2 Gerontology Research Center, Tampere, Finland; 3 Research Centre of Applied and Preventive Cardiovascular Medicine and the Department of Clinical Physiology and Nuclear Medicine, University of Turku and Turku University Hospital, Turku, Finland; 4 Department of Clinical Physiology, Tampere University Hospital and University of Tampere, School of Medicine, Tampere, Finland; 5 Department of Clinical Chemistry, School of Medicine, University of Tampere, Tampere, Finland; 6 School of Health Sciences, University of Tampere, Tampere, Finland; 7 Fimlab laboratories, Tampere, Finland

## Abstract

**Background:**

Human aging is associated with profound changes in one of the major epigenetic mechanisms, DNA methylation. Some of these changes occur in a clock-like fashion, i.e., correlating with the calendar age of an individual, thus providing a new aging biomarker. Some reports have identified factors associated with the acceleration of the epigenetic age. However, it is also important to analyze the temporal changes in the epigenetic age, i.e., the duration of the observed acceleration, and the effects of the possible therapeutic and lifestyle modifications.

**Methods:**

To address this issue, we determined the epigenetic age for a cohort of 183 healthy individuals using blood samples derived from two time points that were 25 years apart (between 15–24 and 40–49 years of age). Additionally, we also determined the epigenetic ages of 119 individuals in a cohort consisting of 90-year-old participants (nonagenarians). These were determined by using the Horvath algorithm based on the methylation level of 353 CpG sites. The data are indicated as the deviation of the epigenetic age from the calendar age (calendar age minus epigenetic age = delta age, ΔAGE). As obesity is often associated with accelerating aging and degenerative phenotypes, the correlation of the body mass index (BMI) with the ΔAGE was analyzed in the following three age groups: young adults, middle-aged, and nonagenarian.

**Results:**

The data showed that BMI is associated with decreased ΔAGE, i.e., increased epigenetic age, in middle-aged individuals. This effect is also seen during the 25-year period from early adulthood to middle age, in which an increase in the BMI is significantly associated with a decrease in the ΔAGE. We also analyzed the association between BMI and epigenetic age in young and elderly individuals, but these associations were not significant.

**Conclusion:**

Taken together, the main finding on this report suggests that association between increased BMI and accelerated epigenetic aging in the blood cells of middle-aged individuals can be observed, and this effect is also detectable if the BMI has increased in adulthood. The fact that the association between BMI and epigenetic age can only be observed in the middle-aged group does not exclude the possibility that this association could be present throughout the human lifespan; it might just be masked by confounding factors in young adults and nonagenarian individuals.

**Electronic supplementary material:**

The online version of this article (doi:10.1186/s13148-016-0301-7) contains supplementary material, which is available to authorized users.

## Background

Aging is characterized by a progressive decline in physiological and cognitive functions. The chronological age of an individual is a natural parameter of choice when predicting the onset of aging-associated diseases and mortality risk. However, due to the innate complexity of aging, the onsets of conditions can dramatically vary between individuals, making chronological age a limited predictor of aging-associated conditions. To address this discrepancy, there have been numerous attempts to establish a universal biomarker for aging, i.e., an attribute that would measure the biological age of an individual [28]. When the establishment of several disease-specific biomarkers has been successful, determination of the biological age of an individual has proven to be a more difficult task. However, despite the unsuccessful attempts, research on aging biomarker candidates has been informative and increased knowledge on changes in various aging-associated functions (e.g., inflammatory cytokines and telomere length) [28].

One of the most recent approaches used to assess the biological age is the utilization of epigenetic mechanisms, namely, DNA methylation. This approach is based on the observations that aging is associated with changes in the DNA methylation levels [6, 12, 18]. More specifically, at the genome-wide level, thousands of methylation-sensitive cytosine bases residing in the CpG (cytosine-phosphate-guanine) sites along DNA are either hyper- or hypomethylated when DNA methylomes of younger and older individuals are compared (reviewed in [31]). Further analysis of the aging-associated CpG sites has revealed that several of them possess an intriguing feature wherein the level of DNA methylation changes in a clock-like fashion, i.e., correlating with the calendar age of an individual [7]. Using several publicly available Illumina 27 and 450 K methylation array datasets, Horvath established an algorithm based on the weighted average of the DNA methylation levels in 353 CpG sites [7]. This algorithm, *the epigenetic clock*, yields an estimate of the individual’s DNA methylation age that generally correlates well with a healthy individual’s calendar age and is claimed to be a successful predictor in most cell types and tissues. The acceleration of epigenetic age can be evaluated by calculating the deviation of calendar age from epigenetic age (ΔAGE). A rapidly increasing number of reports have identified factors and phenotypes that have an accelerating effect on the ticking rate of the epigenetic clock, indicating acceleration of biological aging. Examples of these include infection with HIV [9] or cytomegalovirus [14] and reduced mental and physical fitness in elderly individuals [16]. Additionally, one report demonstrates that accelerated epigenetic aging is associated with a shortened lifespan during follow-up [17]. Base level of ΔAGE has been shown to be fixed before adulthood after which there is very little variation [13].

Obesity has several adverse effects on the body, leading to increased atherosclerosis, tumorigenesis, neurodegeneration, and type 2 diabetes, i.e., conditions that are classical features of advanced aging (reviewed in [4]). It has also been demonstrated that obesity is associated with a shortened lifespan [4]. Interestingly, although the health risks associated with overweight and obesity are well established [23], there is some controversy surrounding the issue of how this applies to older individuals. According to the obesity paradox, obese elderly people have better outcomes in conditions such as hypertension, coronary artery disease, peripheral artery disease, and congestive cardiac failure than normal-weight individuals of the same age [2]. Additionally, significant decreases in the mortality of obese individuals with heart failure have been reported compared to their normal-weight counterparts [27].

To characterize the significance of obesity in epigenetic aging, we analyzed the association between BMI and epigenetic age in three age groups: young adults, middle-aged, and nonagenarian individuals.

## Methods

### Study populations

The data from two different study populations were studied to analyze the relationship between the BMI and epigenetic age in three age groups: young, middle-aged, and nonagenarian individuals (Table 1). The young and middle-aged groups consisted of matched samples (*n* = 183) with data from two time points that were 25 years apart, and the nonagenarian group included 119 individuals (women *n* = 87, men *n* = 32) from another study. For the sake of clarity, the age groups are referred to as YFS1986 (the young group), YFS2011 (the middle-aged group), and the V90+ (the nonagenarian group) (for a more detailed description of the sample populations, see Table 1). The YFS1986 and YFS2011 age groups consisted of the individuals from the Cardiovascular Risk in Young Finns Study (YFS), an ongoing follow-up study with data collected at several time points from childhood to middle age (for a more detailed description of the YFS cohort, see [25]). The YFS1986 and YFS2011 samples collected in 1986 and 2011, respectively, included 183 individuals (women *n* = 111, men *n* = 72). The sample size was determined by the availability of BMI and DNA methylation data. The Young Finns Study was approved by the local ethics committees (the University Hospitals of Helsinki, Turku, Tampere, Kuopio, and Oulu) and conducted according to the guidelines of the Declaration of Helsinki. All participants gave their written informed consent.

**Table 1:** Data summary of the cohorts used in this study. Data includes mean, standard deviation, and range of calendar age in years, epigenetic age in years, ΔAGE (difference between chronological age and epigenetic age) in years, and BMI in kg/m^2^

	YFS1986^a^ (*n* = 183) Young adults	YFS2011^b^ (*n* = 183) Middle-aged	Vitality 90+ (*n* = 119) Nonagenarians
Calendar age			
Mean	19.2	44.2	90
Standard deviation	3.25	3.25	0^c^
Range	15–24	40–49	90–90^c^
Epigenetic age			
Mean	17.5	43.6	76.3
Standard deviation	4.36	4.48	6.17
Range	4.87–29.71	33.23–61.37	62–101
ΔAGE			
Mean	1.74	0.60	13.71
Standard deviation	2.96	3.70	6.17
Range	−6–10.35	−12.53–9.31	−11–28
BMI			
Mean	21.32	26.13	26.38
Standard deviation	2.74	4.58	4.86
Range	16.75–34.32	18.87–45.18	13.67–38.26

^a^Young Finns Study, samples collected in 1986

^b^Young Finns Study, samples collected in 2011

^c^All individuals in the Vitality 90+ Study were 90 years old

The contribution for the nonagenarian age group was provided by the participants of the V90+ study, an ongoing prospective population-based study consisting of individuals aged 90 years and older and living in the city of Tampere, Finland (for the description of the recruitment and characterization of the participants, see [5]). All individuals provided written informed consent, and the research protocol followed the guidelines of the Declaration of Helsinki. The V90+ study samples were collected in 2010. The mean ages of the individuals in the three age groups were 19.2 (YFS1986), 44.2 (YFS2011), and 90.0 (V90+) years. All individuals included in this study were selected from the YFS and Vitality 90+ study populations based on the availability of the BMI and DNA methylation array data.

### Sample collection

In the Vitality 90+ study, blood samples were drawn into EDTA tubes by properly trained medical during home visits. All the samples were collected between 8 and 12 a.m. and leukocyte separation was performed directly with a Ficoll-Paque density gradient (Ficoll-Paque™ Premium, cat no. 17-5442-03, GE Healthcare Bio-Sciences AB, Uppsala, Sweden). Following the separation, the peripheral blood mononuclear cell (PBMC) layers were collected, and DNA was extracted using the spin protocol for the QIAamp DNA Mini kit (Qiagen, CA, USA), according to the manufacturer’s instructions. The DNA concentration was assessed using a Qubit dsDSNA HS assay (Invitrogen, Eugene, OR, USA), and the quality was checked with a NanoDrop (Thermo Scientific, USA). Without the availability of separated PBMCs, whole blood leukocytes (WBL) were used instead of the YFS samples. The WBL DNA of the YFS cohorts was obtained from blood samples stored in EDTA using a Wizard® Genomic DNA Purification Kit (Promega Corporation, Madison, WI, USA) according to the manufacturer’s instructions (for more detailed sample collection details of YFS, see Nuotio et al. [21]). The quality and concentration of the WBL DNA was assessed using a NanoDrop. For the YFS1986 samples, the intactness of the DNA was assessed by performing PCR for the amelogenin gene (*AMELX* and *AMELY*).

### DNA methylation array

Methylation profiling of the samples in Vitality 90+ study and in YFS2011 was performed at the Institute for Molecular Medicine Finland (FIMM) Technology Centre, University of Helsinki. DNA (1 μg) was used for the bisulfite conversion with the EZ-96 DNA Methylation Kit (Zymo Research, Irvine, CA, USA), according to the manufacturer’s protocol. Converted DNA (4 μl) was whole-genome amplified, enzymatically fragmented, and hybridized to the Infinium HumanMethylation450 BeadChip (Illumina Inc., CA, USA), covering sequences for the analysis of 485000 CpG sites. Samples were applied in the BeadChips in random order. Results were obtained using the iScan reader (Illumina Inc., San Diego, CA, USA). Methylation profiling of the samples in YFS from 1986 was performed at Helmholtz Zentrum, München, Germany, using the same methodology. The quality of the DNA methylation data was carefully inspected by standard examinations with principal component analysis (PCA) and visualizations with boxplots, density plots, and dotplots. The possible bias of batch effects was later addressed by normalizing the DNA methylation data using the BMIQ function that is implemented in the DNAmAge algorithm, as described below. The overall quality control was supported by technical replicates and gender prediction based on the X chromosome methylation (Additional file 1: Fig. S1).

### Quantification of the epigenetic age and deviation of the calendar age

Obtained probe intensities were transformed to *β* values with a standard equation in which *β* is the ratio of the methylated probe (m) intensities to the overall intensities (m + u + α, where α is the constant offset, 100, and u is the unmethylated probe intensity). The resulting *β* values ranged from 0 (completely unmethylated, 0) to 1 (fully methylated, 100%). Principal component analysis (PCA) and visualization of the methylation intensity values were used to assess the quality control. *β* values of the selected probes were used as the input for the calculating the epigenetic age (https://dnamage.genetics.ucla.edu/home) [7]. Normalization of the batch effects was performed with the BMIQ function implemented in the DNAmAge algorithm. The deviation of the estimated epigenetic age from the actual calendar age was calculated by subtracting the former from the latter, yielding ΔAGE (calendar age minus epigenetic age = delta age, ΔAGE).

### Correlation analysis of the BMI and ΔAGE

The association between the BMI and ΔAGE was investigated using Spearman’s rank correlation separately in each age group. Confounding factors in the association between the BMI and ΔAGE in the middle-aged group were addressed with a linear regression model (stepwise, criteria = PIN(0.05) POUT(0.10)). The following available variables were used in the regression model to predict the ΔAGE: BMI, alcohol consumption, smoking, and gender. The prevalence of chronic diseases and medications was well below 10%; as a result, they were not included in the model.

## Results

**Fig. 1:**
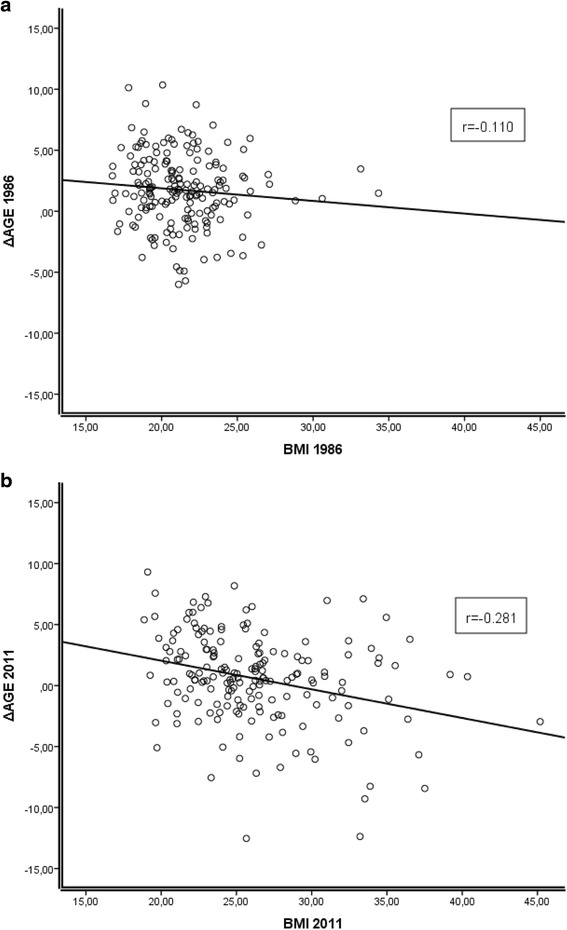
**a** Association between ΔAGE (difference between chronological age and epigenetic age in years) and BMI in young adults of the Young Finns Study (mean age of 19.2 years). Association is non-significant (*r* = −0.110, p = 0.138). **b** Association between ΔAGE and BMI in middle-aged individuals of the Young Finns Study (mean age of 44.2 years, follow-up from Fig. 1a). Correlation is significant (*r* = *−*0.281, *p* = 0.0001)

**Fig. 2:**
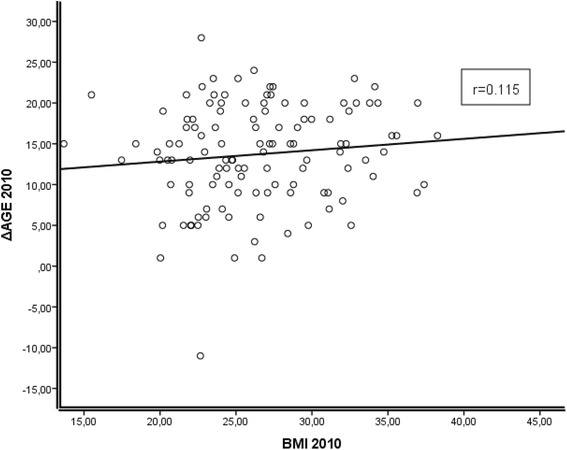
Association between ΔAGE and BMI in nonagenarian individuals of the Vitality 90+ Study (Age of 90 years). Correlation is non-significant (*r* = 0.115, *p* = 0.211). However, it is noteworthy that the trend of correlation is opposite to corresponding ones of the young adults and middle-aged (Fig. 1a, b). This could be an indication of obesity paradox where higher BMI is beneficial for the elderly individuals

The mean BMI levels of the young adults, middle-aged, and nonagenarians were 21.32, 26.13, and 26.38 kg/m^2^, respectively. The mean epigenetic ages of these subpopulations were 17.5, 43.6, and 76.3 years (Table 1). The mean deviations of the epigenetic age from the calendar age, the ΔAGE, were 1.74, 0.60, and 13.71 years, respectively. The association between the BMI and ΔAGE in young and middle-aged individuals was analyzed with matched samples at 25-year interval time points (samples collected in 1986 and 2011), and the mean calendar ages of the individuals in these consecutive time points were 19.2 and 44.2 years, respectively. The WBLs were used as a source of the DNA. The correlation of ΔAGE with BMI is shown in Fig. 1 (panel A, young adults; panel B, middle-aged). In the young adult subpopulation, the samples were collected in 1986, and there was no significant association between the ΔAGE and BMI (*r* = −0.110, *p* = 0.138), indicating that BMI did not alter the ticking rate of the epigenetic clock as these individuals entered into adulthood. The middle-aged subpopulation, however, had a significant inverse correlation between BMI and ΔAGE (*r* = *−*0.281, *p* = 0.0001), suggesting that higher BMI is associated with acceleration of epigenetic aging in middle-aged individuals. The gender did not affect this correlation; the result was also significant when performed according to gender (Additional file 2: Table S1 and Additional file 3: Fig. S2). Confounding factors were addressed with a linear regression model, and BMI was the single best predictor of the ΔAGE (Additional file 4: Table S2). Within the nonagenarian subpopulation, there was no significant association between the ΔAGE and BMI (*r* = 0.115, *p* = 0.211, and Fig. 2).

**Fig. 3:**
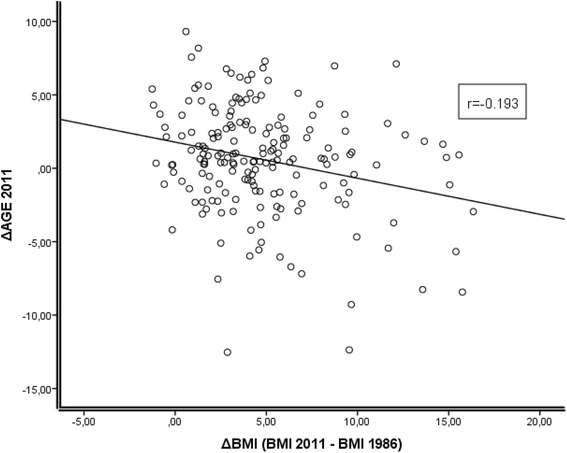
Association between ΔAGE and change in BMI in a 25-year follow-up in middle-aged individuals of the Young Finns Study. Correlation is significant (*r* = −0.193, *p* = 0.009), indicating that increase in BMI has had an accelerating effect on epigenetic aging

To understand the long-term effects of BMI, we analyzed the association between the change in BMI over a 25-year time range, as well as the ΔBMI (BMI in 2011 minus BMI in 1986) and ΔAGE in middle-aged individuals. Figure 3 shows a significant inverse correlation between the change in the BMI and ΔAGE (*r* = −0.193, *p* = 0.009), suggesting that the individual’s current BMI status is not the only factor affecting the epigenetic age and that increased epigenetic age is instead an outcome of weight gain over time. It should be noted, however, that only a few individuals showed a decrease in BMI between these years. As a result, this association mainly represents the effects of increasing BMI over time.

## Discussion

Aging is an extremely complex and heterogenic phenomenon that is associated with a general decrease in several cellular and organ functions. Due to this heterogeneity, the actual calendar age of an individual seems to tell very little about what is actually going on inside the tissues and cells in terms of the aging process itself. This discrepancy has paved the way for the development of an aging biomarker that would estimate the state of one’s aging process better than chronological age. One of the most recent approaches to identifying aging biomarkers is the epigenetic clock, which is based on the quantification of methylation states of the cytosine bases in the DNA. Since the discovery of the concept of an epigenetic clock [7], numerous studies have attempted to explain the factors involved in determining of one’s epigenetic age.

Obesity is linked to aging as it is associated with certain age-related conditions such as diabetes [1] and shortening of the telomeres [29]. The role of BMI in the determination of life-span has been studied extensively. For example, analysis of the follow-up data of 900000 individuals (mean recruitment age 46 years, and mean age at death 67 years) indicated that the mortality was lowest at a BMI of approximately 22.5−25 kg/m^2^ and higher; each 5 kg/m^2^ increase in the BMI was associated with a 30% higher mortality and association could also be seen below the 22.5−25 kg/m^2^ range [24]. Additionally, the length of the time spent in a high BMI state and lifetime peak BMI can have a positive association with mortality [19]. Obesity associated epigenetic changes have also been looked into. Some studies have reported that increased BMI is associated with hypermethylation of several CpG sites within the *HIF3A* gene [26] as well as hypermethylation of singe CpG site in *TRIM3* gene and hypomethylation of single CpG site in *UBASH3A* gene [30]. Additionally, there are reports regarding association between global methylation levels of Alu–elements and BMI [20], and association between obesity and DNA methylation levels in leptin (LEP) and adiponectin (ADIPOQ) genes [11]. These obesity-related DNA methylation changes do not seem to overlap those seen in aging-associated changes [12, 18]. However, instead of focusing on single CpG site hypo- or hypermethylation events, we wanted to explore the relationship between overweight, as represented by BMI and the epigenetic age, an aging biomarker calculated using the methylation status of multiple CpG sites. In this study, we investigated the association between BMI and epigenetic age in the blood cells of three different age groups, the young adults, the middle-aged, and the nonagenarians. We found that BMI is not associated with epigenetic aging in young adults, in the age range from 15 to 24, nor in nonagenarians, the individuals aged 90 years. In the middle-aged subpopulation, however, the BMI was inversely correlated with individual’s ΔAGE. In addition, also the change in BMI over 25 years was similarly inversely correlated with one’s ΔAGE at the later time point.

We did not observe the association between BMI and the acceleration of the epigenetic aging in young adults (Fig. 1a). It has been shown that epigenetic clock of the children is ticking relatively faster than that of adults because of the active, ongoing growth period [7]. Thus, organismal growth accompanied with the high number of cell divisions leads to logarithmic ratio of epigenetic age and calendar age. After the growth period is over, ticking rate of the epigenetic clock slows down to constant and increases linearly with calendar age [7]. Therefore, the finding that increased BMI has not accelerated the epigenetic aging of the young adults (mean age of 19.2 years) suggests that obesity-induced acceleration of the epigenetic age takes place over time, and this population has not been exposed to it long enough so that the effect could be observed. It is also possible that the effect of increased BMI is minor when compared to epigenetic aging induced by normal growth, and therefore cannot be distinguished.

Epigenetic aging of the older study populations have also been looked into. It has been shown that extremely old individuals, such as centenarians, possess significantly lower epigenetic ages than what their calendar age would suggest [10]. The older individuals in our study were represented by the nonagenarians and similar trend was observed, their epigenetic age being on average 13.7 years lower than their calendar age. This is probably due to the opposite of what happens during the active growth period, as the regenerative capacity of the body diminishes [22] and the epigenetic clock does not keep up. Additionally, the DNA methylomes of nonagenarians have been exposed to environmental factors for decades and global aging-associated hypo- and hypermethylation of the CpG sites could potentially distort the accuracy of DNAmAge algorithm. However, it is also possible that the nonagenarians are selected in a way that those individuals with higher epigenetic ages have already died, and those with relatively lower epigenetic ages are overrepresented in our study population.

We did not observe significant association between individual’s BMI and epigenetic age in the nonagenarian study population. Interestingly, however, the correlation trend of these variables was opposite to that seen in the younger and middle-aged individuals, meaning that higher BMI corresponded to higher ΔAGE, and thus lower epigenetic age. Although not significant, this discrepancy could be a manifestation of obesity paradox which suggests that overweight could be in fact beneficial in very old individual, as opposed to young people for whom it is a risk factor for morbidity and mortality (reviewed in [2]). In nonagenarians, low BMI, waist-to-hip ratio, and waist circumference have been shown to predict mortality [15].

The main finding in this report suggests that increased BMI is associated with accelerated epigenetic aging in the blood cells of middle-aged individuals (Fig. 1b). The mechanisms connecting the numerous metabolic changes induced by obesity to the process of DNA methylation can only be proposed at present. Additionally, the role of the obesity-induced degenerative disease processes cannot be excluded. However, as the individuals of the affected subpopulation are not very old (still <50 years of age), their role is probably minor. Another key finding of this study is the observation that the BMI changes in the 25-year range, from early adulthood to middle age, can accelerate the ticking rate of epigenetic clock, indicating that the clock itself is adjustable (Fig. 3). It should be noted that only a few individuals in this study demonstrated a decrease in the BMI during the 25-year follow-up; therefore, its effect could not be analyzed. This information would be decisive for understanding the basic mechanisms of the BMI/epigenetic clock correlation. Implications of accelerated epigenetic aging are beyond the scope of this article, but according to literature, these epigenetic alterations could be the manifestations of accumulated DNA damage over time [3], establishing the link between epigenetic and calendar age. If this is the case, it seems implausible that epigenetic changes could be reversed by lowering the BMI. However, additional follow-up studies are needed to investigate the association between long-term weight reduction and epigenetic aging. Understanding this association could offer important information for planning various weight reduction or caloric restriction protocols.

Horvath et al. [8] observed that obesity accelerates epigenetic aging of the human liver. However, in contrast to the data presented here, they did not observe this phenomenon in DNA derived from blood cells. As the methodology (source of DNA, methylation assay, and calculation algorithm) is exactly the same, it is likely that this discrepancy is due to the differences in the study cohorts. Our study cohort is very homogeneous, consisting of individuals with the same ethnic and genetic backgrounds, which may reduce the effect of possible confounders. Moreover, the possibility for follow-up analysis increases the reliability of the analysis. It is evident that very large, well-characterized population cohorts are required to reveal the factors that influence the ticking rate of the epigenetic clock. The limitations of our study include the restricted sample size and slightly differing source of DNA between the YFS (whole blood) and V90 (PBMC) study populations. However, based on our earlier results, these limitations do not seem to have a major effect [13].

## Conclusions

Overall, our results suggest that accelerated epigenetic aging could be due to the active growth or regeneration of tissues. This is evident in the children undergoing the active growth phase, when their epigenetic clock ticks faster [7]. In elderly individuals, the regeneration capacity of the tissues and stem cells decreases, leading to slower ticking of the clock. Somewhere, in between these two age periods, when the baseline epigenetic aging is linear, adding fat tissue could be regarded as growth. Hence, the underlying epigenetic control mechanisms might be the same as in childhood. The fact that the association between BMI and epigenetic age can only be observed in the middle-aged group, does not exclude the possibility that this association could be present throughout the human lifespan; it might just be masked by confounding factors in young adults and nonagenarian individuals.

Regarding future studies, it would be interesting to investigate whether increased muscle mass is associated with accelerated epigenetic aging, as it also involves proliferation of the tissues outside of the active growth phase. Also, further studies are needed to establish the more precise mechanisms that operate behind the observed association between epigenetic age and BMI.

## Additional files

Additional file 1: Figure S1. The quality control of the DNA methylation of old DNA samples. YFS1986 samples (young adults) were collected 30 years ago, and the methylation profiling was performed in different facility than others. To ensure that these samples did not include technical biases, we compared the mean methylation of X chromosome in YFS1986 (*X axis*) and YFS2011 (*Y axis*) follow-up samples. These two cohorts cannot be distinguished from the plot, and only gender stands out, as expected in the case of DNA methylation. (DOCX 59 kb)
Additional file 2: Table S1. Gender-specific correlations of ΔAGE and BMI in three age groups: the young adults (BMI1986 and ΔAGE 1986), the middle-aged (BMI 2011 and ΔAGE 2011), and the nonagenarians (BMI 2010 and ΔAGE 2010). The correlation is significant only in middle-aged males and females. (XLSX 10 kb)
Additional file 3: Figure S2. Gender-specific correlations of ΔAGE and BMI in middle-aged individuals. In panel **A**, scatterplot of ΔAGE and BMI in middle-aged female individuals (*r*=−0265, *p*=0.05). In panel **B**, scatterplot of ΔAGE and BMI in middle-aged male individuals (*r*=−0.270, *p*=0.022). (DOCX 51 kb)
Additional file 4: Table S2. The linear regression model explaining the variation in ΔAGE of middle-aged adults. We used BMI, gender, smoking, and alcohol consumption as predictors. Of these, the BMI was the best independent predictor of ΔAGE (*p*=0.002). The regression method was stepwise, criteria=PIN(0.05) POUT(0.10). (DOCX 14 kb)
