# Supplementary material for: Obesity accelerates epigenetic aging in middle-aged but not in elderly individuals
Source: Clin Epigenetics. 2017 Feb 14;9:20. doi: 10.1186/s13148-016-0301-7 (PMC5310016; doi:10.1186/s13148-016-0301-7)
Supplement: Additional file 1: Figure S1. — The quality control of the DNA methylation of old DNA samples. YFS1986 samples (young adults) were collected 30 years ago, and the methylation profiling was performed in different facility than others. To ensure that these samples did not include technical biases, we compared the mean methylation of X chromosome in YFS1986 (X axis) and YFS2011 (Y axis) follow-up samples. These two cohorts cannot be distinguished from the plot, and only gender stands out, as expected in the case of DNA methylation. (DOCX 59 kb) [file 13148_2016_301_MOESM1_ESM.docx]

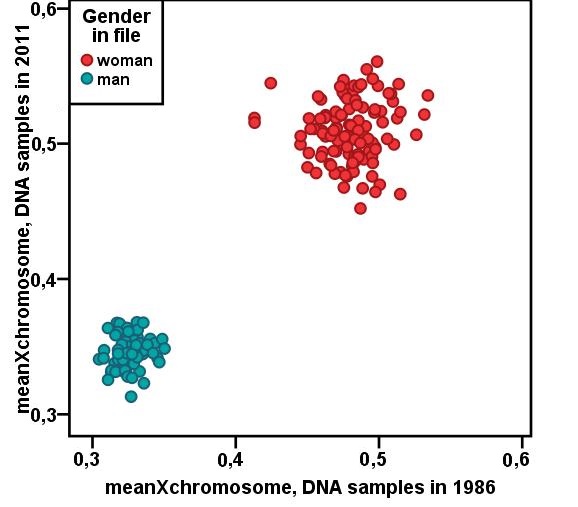


**Additional file 1: Figure S1.** The quality control of the DNA methylation of old DNA samples. YFS1986 samples (young adults) were collected 30 years ago and the methylation profiling was performed in different facility than others. To ensure that these samples did not include technical biases we compared the mean methylation of X chromosome in YFS1986 (X-axis) and YFS2011 (Y-axis) follow-up samples. These two cohorts cannot be distinguished from the plot and only gender stands out, as expected in the case of DNA methylation.
