# Supplementary material for: Obesity accelerates epigenetic aging in middle-aged but not in elderly individuals
Source: Clin Epigenetics. 2017 Feb 14;9:20. doi: 10.1186/s13148-016-0301-7 (PMC5310016; doi:10.1186/s13148-016-0301-7)
Supplement: Additional file 3: Figure S2. — Gender-specific correlations of ΔAGE and BMI in middle-aged individuals. In panel A, scatterplot of ΔAGE and BMI in middle-aged female individuals (r=−0265, p=0.05). In panel B, scatterplot of ΔAGE and BMI in middle-aged male individuals (r=−0.270, p=0.022). (DOCX 51 kb) [file 13148_2016_301_MOESM3_ESM.docx]

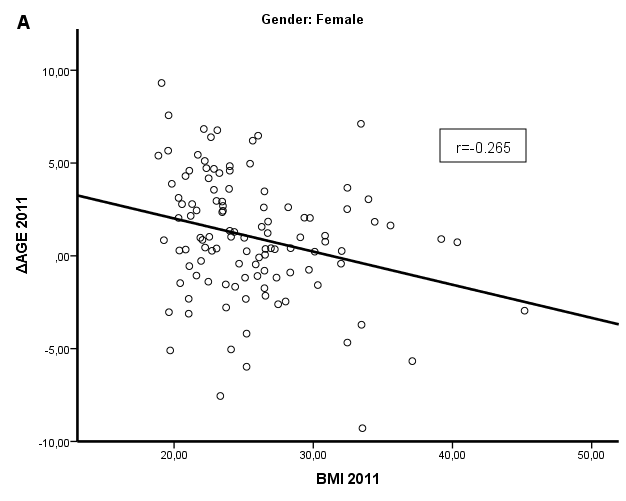


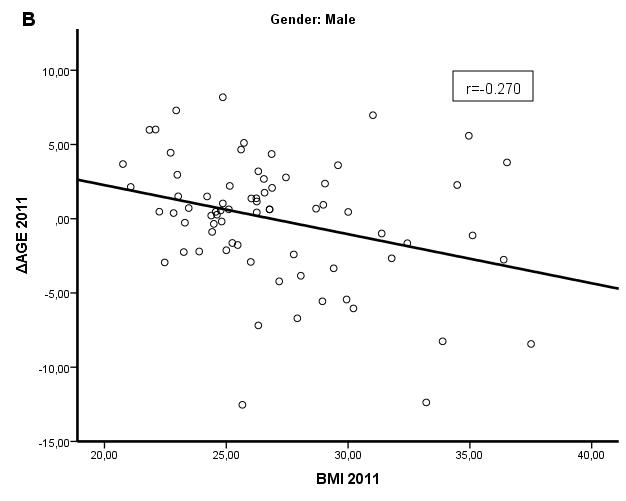


**Additional file 3: Figure S2.** Gender-specific correlations of ΔAGE and BMI in middle-aged individuals. In panel **A**, scatterplot of ΔAGE and BMI in middle-aged female individuals (r=-0265, p=0.05). In panel **B**, scatterplot of ΔAGE and BMI in middle-aged male individuals (r=-0.270, p=0.022).
