# Supplementary material for: Obesity accelerates epigenetic aging in middle-aged but not in elderly individuals
Source: Clin Epigenetics. 2017 Feb 14;9:20. doi: 10.1186/s13148-016-0301-7 (PMC5310016; doi:10.1186/s13148-016-0301-7)
Supplement: Additional file 4: Table S2. — The linear regression model explaining the variation in ΔAGE of middle-aged adults. We used BMI, gender, smoking, and alcohol consumption as predictors. Of these, the BMI was the best independent predictor of ΔAGE (p=0.002). The regression method was stepwise, criteria=PIN(0.05) POUT(0.10). (DOCX 14 kb) [file 13148_2016_301_MOESM4_ESM.docx]

**Additional file 4: Table S2.** The linear regression model explaining the variation in ΔAGE of middle-aged adults. We used BMI, gender, smoking and alcohol consumption as predictors. Of these, the BMI was the best independent predictor of ΔAGE (p=0.002). The regression method was stepwise, criteria=PIN(0.05) POUT(0.10).

|  |  |  |  |  |  |  |
| --- | --- | --- | --- | --- | --- | --- |
| **Regression** |  |  |  |  |  |  |
|  |  |  |  |  |  |  |
| Variables Entered/Removed^a^ | | | |  |  |  |
| Model | Variables Entered | Variables Removed | Method |  |  |  |
| 1 | BMI 2011 |  | Stepwise (Criteria: Probability-of-F-to-enter <= ,050, Probability-of-F-to-remove >= ,100). |  |  |  |
| a. Dependent Variable: ΔAGE 2011 | | | |  |  |  |
|  |  |  |  |  |  |  |
| **Model Summary** | | | | |  |  |
| Model | R | R Square | Adjusted R Square | Std. Error of the Estimate |  |  |
| 1 | ,305^a^ | 0,093 | 0,084 | 3,55979 |  |  |
| a. Predictors: (Constant), BMI 2011 | | | | |  |  |
|  |  |  |  |  |  |  |
| ANOVA^a^ | | | | | | |
| Model | | Sum of Squares | df | Mean Square | F | Sig. |
| 1 | Regression | 127,590 | 1 | 127,590 | 10,069 | ,002^b^ |
|  | Residual | 1241,865 | 98 | 12,672 |  |  |
|  | Total | 1369,454 | 99 |  |  |  |
| a. Dependent Variable: ΔAGE 2011 | | | | | | |
| b. Predictors: (Constant), BMI 2011 | | | | | | |
|  |  |  |  |  |  |  |
| Coefficients^a^ | | | | | | |
| Model | | Unstandardized Coefficients | | Standardized Coefficients | t | Sig. |
|  |  | B | Std. Error | Beta |  |  |
| 1 | (Constant) | 6,774 | 2,001 |  | 3,385 | 0,001 |
|  | BMI 2011 | -0,236 | 0,074 | -0,305 | -3,173 | 0,002 |
| a. Dependent Variable: ΔAGE 2011 | | | | | | |
|  |  |  |  |  |  |  |
| Excluded Variables^a^ | | | | | | |
| Model | | Beta In | t | Sig. | Partial Correlation | Collinearity Statistics |
|  |  |  |  |  |  | Tolerance |
| 1 | Gender | -,189^b^ | -1,984 | 0,050 | -0,198 | 0,993 |
|  | Smoking | ,031^b^ | 0,323 | 0,747 | 0,033 | 1,000 |
|  | Alcohol consumption | -,060^b^ | -0,617 | 0,538 | -0,063 | 1,000 |
| a. Dependent Variable: ΔAGE 2011 | | | | | | |
| b. Predictors in the Model: (Constant), BMI 2011 | | | | | | |
